# Tailoring optical response of MXene thin films

**DOI:** 10.1515/nanoph-2024-0769

**Published:** 2025-04-03

**Authors:** Jeffrey Simon, Kyu Ri Choi, Stefano Ippolito, Ludmila Prokopeva, Colton Fruhling, Vladimir M. Shalaev, Alexander V. Kildishev, Yury Gogotsi, Alexandra Boltasseva

**Affiliations:** Elmore Family School of Electrical and Computer Engineering, Birck Nanotechnology Center and Purdue Quantum Science and Engineering Institute, 311308 Purdue University, West Lafayette, IN, 47907, USA; Research Institute for Nanoscale Science & Technology, Chungbuk National University, Cheongju, Chungbuk, 28644, Republic of Korea; A. J. Drexel Nanomaterials Institute and Department of Materials Science and Engineering, Drexel University, Philadelphia, PA, 19104, USA; School of Materials Engineering, Purdue University, West Lafayette, IN, 47907, USA

**Keywords:** MXenes, 2D materials, plasmonics, solution processing, optical tunability, ENZ properties

## Abstract

Due to their attractive optical properties, 2D MXenes have garnered interest in nanophotonic and optoelectronic applications. However, tuning their properties typically requires the iterative synthesis of MXenes with a specific set of properties, such as the absorption band position, electronic conductivity, and dielectric constant. We demonstrate how to tailor the optical properties of MXene thin films over a broad 1500-nm wavelength range by mixing different ratios of highly conductive Ti_3_C_2_T_*x*_ with poorly conductive Nb_2_CT_*x*_. By changing the MXene film composition, the epsilon-near-zero (ENZ) point, where the optical properties transit from dielectric to metallic, was varied in the spectral range from 1.1 to 2.6 µm. Additionally, we observed a reduction in absorption in some compositions compared to the absorption of the pure MXene films. Compared to other methods, this approach enables simple and continual tuning of MXene optical properties without requiring multiple time-consuming synthesis steps.

## 1 Introduction

MXenes, two-dimensional transition-metal carbides, nitrides, and carbonitrides, have the general formula M_*n*+1_X_*n*_T_*x*_, where M represents an early transition metal, X is carbon and/or nitrogen, *n* ranges from 1 to 4, and T_*x*_ represents the surface terminations. The surface terminations are commonly a mixture of –O, –F, and –OH groups when the standard wet-chemical etching process is used [1], [2]. The family of MXenes includes dozens of currently synthesized single- and multi-transition metal compositions and supports a wide variety of optical properties, including dielectric, metallic, and theoretically predicted topological insulator behaviors [3], [4], [5], leading to a broad range of applications. For instance, MXenes have been utilized as substrates for surface-enhanced Raman scattering (SERS) [6], saturable absorber elements in mode-locked lasers [7], [8], [9], [10], absorber materials in photodetectors [11], [12], and a broadband metamaterial absorber [13].

**Figure 1:**
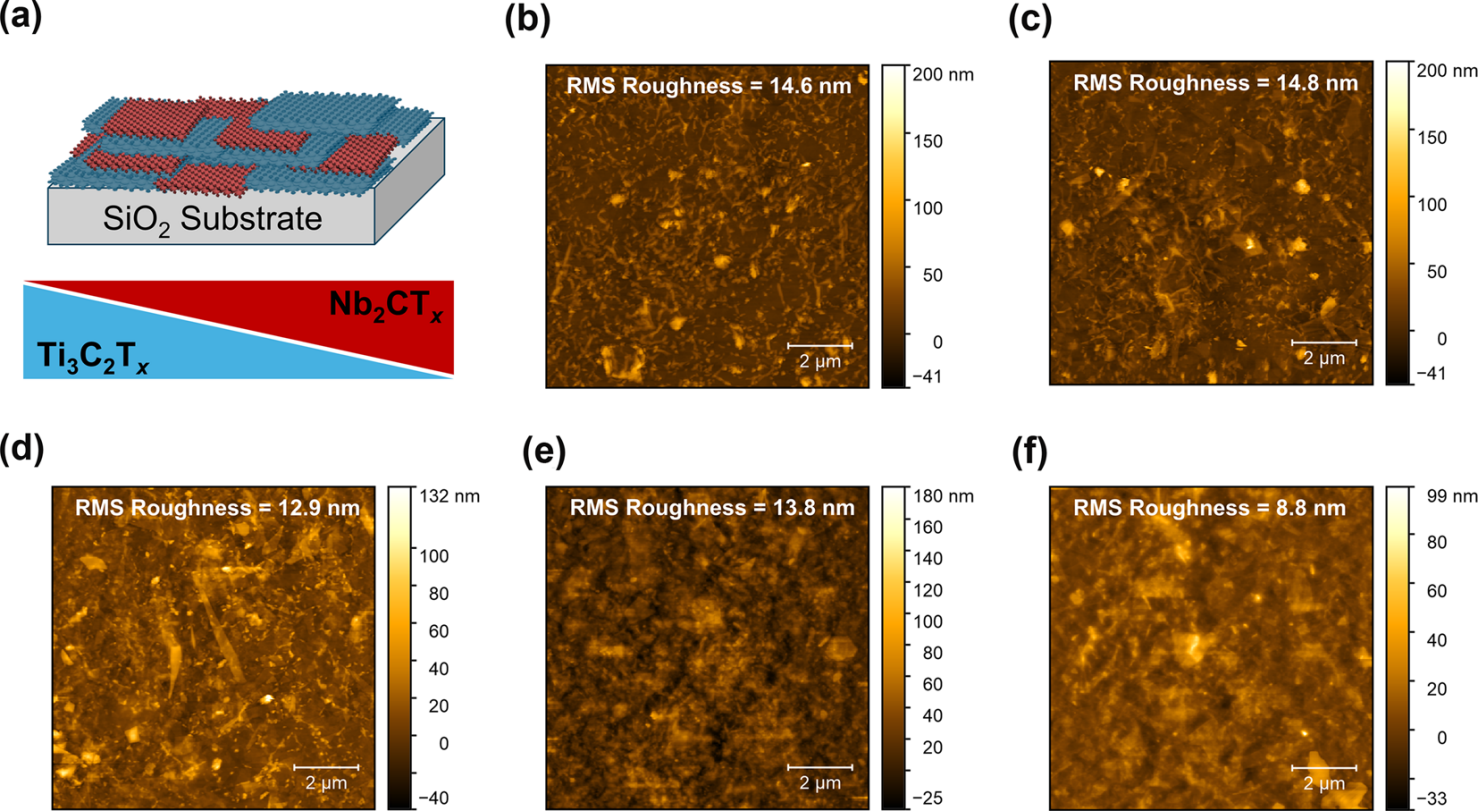
Randomly stacked MXene films. (a) A schematic representation of (top) a mixed film of Ti_3_C_2_T_*x*_ and Nb_2_CT_*x*_ MXene flakes with (bottom) concentrations that can be continuously varied. AFM images of (b) Ti_3_C_2_T_*x*_, (c) Ti_3_C_2_T_*x*_(75):Nb_2_CT_*x*_(25), (d) Ti_3_C_2_T_*x*_(50):Nb_2_CT_*x*_(50), (e) Ti_3_C_2_T_*x*_(25): Nb_2_CT_*x*_(75), and (f) Nb_2_CT_*x*_ mixed-MXene films.

MXene films consist of a stacked layer-like arrangement of flakes (Figure 1a). They are usually deposited onto substrates using various solution-based processes, including spin, dip, or spray coating [14]. The resulting film’s optical properties depend on the flake’s electronic band structure, average size, and overall architecture (i.e., the physical arrangement of flakes within the film) [15]. Importantly, the straightforward deposition methods enable the formation of thin and homogeneous films of both optically dielectric and metallic MXenes without high-temperature steps, making MXenes promising and easy-to-process materials for applications in optical devices.

The success of any material for device design relies on the tunability of its optical properties, which is an active area of research for MXenes. This includes the synthesis of MXenes with different transition metals to discretely tune their optical properties [16]. Furthermore, continuous tunability can be achieved through solid-solution MXenes by preparing the MAX phase with a desired ratio of two transition metal elements. Nevertheless, the optimization process is complex, requiring the synthesis of new MAX phases for each iteration [17]. While the flake size and film thickness are also shown to affect the optical properties [13], [15], the realization of large-scale and tailorable MXene films has not been reported. Other techniques for optical control that rely on the selection of surface terminations through annealing, molten-salt etching, and dynamic techniques are effective but have limited tunability range, issues with environmental stability, and affect the flake morphology [18], [19], [20], [21], [22].

This work introduces a simple approach to continuously tailor the optical properties through mixed-MXene films by precisely controlling their composition ratios (Figure 1a). Ti_3_C_2_T_*x*_ has an epsilon-near-zero (ENZ) point in the near-infrared (NIR), where the optical properties transition from dielectric to metallic and the real part of the dielectric permittivity, ℜ $\left(\varepsilon \right)$ , crosses zero. The ENZ point is particularly interesting for optical applications since it has been shown to support unique propagating modes and enhance optical nonlinearities through the slow-light effect [23], [24]. In contrast to Ti_3_C_2_T_*x*_, Nb_2_CT_*x*_ acts as a dielectric across a broad optical spectrum. By combining these two MXenes, the film’s optical properties could be tuned across the visible and short-wave infrared (SWIR) spectra. In addition to variable angle spectroscopic ellipsometry (VASE), the optical responses of the fabricated mixed-MXene films were characterized by UV-visible (VIS)-infrared (IR) reflection, transmission, and absorption spectroscopy. We found that as the concentration of Nb_2_CT_*x*_ increased, the mixed-MXene films still exhibited metallic properties while their losses decreased. By changing the film composition, we observed the dramatic tuning of both the ENZ point and the plasma frequency, with respective redshifts of 1,500 nm and 2 eV.

## 2 Results

Five mixed-MXene thin films were fabricated by spray coating dispersions having different mass ratios of Ti_3_C_2_T_*x*_ and Nb_2_CT_*x*_ onto fused silica substrates (See Methods). This formed randomly stacked films (Figure 1a), with an average thickness of 30 nm (see S1) and varied morphology with respect to the composition of the constituent MXenes (Figure 1b–f). The majority of Ti_3_C_2_T_*x*_ flakes were single layers with a lateral size typically ranging from 5-10 µm [25], while Nb_2_CT_*x*_ flakes had lateral dimensions 5–10 times smaller and a higher percentage of few-layer nanosheets [26]. While each film had a similar RMS surface roughness on the scale of 10 nm, the films with higher Ti_3_C_2_T_*x*_ ratios exhibited more fine wire-like features, representing wrinkles and ripples. Since both the spatial scale of the film roughness and flake thickness were deeply subwavelength, effective media behavior is expected [27].

**Figure 2:**
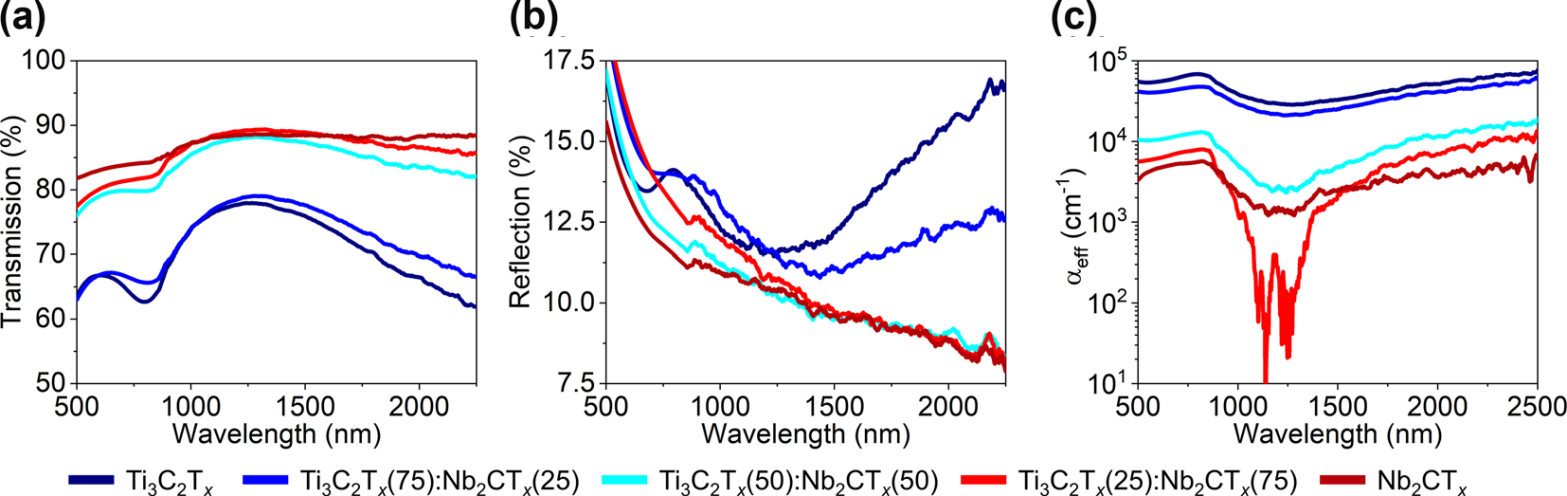
UV-VIS-IR spectroscopy measurements. (a) Transmission and (b) reflection spectra of Ti_3_C_2_T_*x*_: Nb_2_CT_*x*_ mixed-MXene. (c) Absorption coefficients of Ti_3_C_2_T_*x*_: Nb_2_CT_*x*_ mixed-MXene films.

A comprehensive study to investigate the linear optical properties of mixed-MXene films was performed, focusing on transmission enhancement and absorption reduction as the film composition changed. For films with higher Ti_3_C_2_T_*x*_ concentrations, both the transmission and absorption spectra exhibited an increase in resonance strength near 800 nm (Figure 2a–c). Additionally, mixed films with higher Nb_2_CT_*x*_ concentrations exhibited a pronounced improvement in optical transmission and a two-order-of-magnitude decrease in absorption across certain regions of the measured spectral range. This trend indicates that the inclusion of Nb_2_CT_*x*_ contributed to the reduction in optical losses, potentially through the changes in the electronic and structural properties of the mixed films. An anomalous yet intriguing result was the remarkably low absorption observed in the film of Ti_3_C_2_T_*x*_(25):Nb_2_CT_*x*_(75) between 750 and 1,500 nm, exhibiting losses lower than that of the pure films of Ti_3_C_2_T_*x*_ or Nb_2_CT_*x*_ (Figure 2c).

Additionally, films with higher Ti_3_C_2_T_*x*_ concentrations exhibit a steeper slope in the reflection spectrum between 1,300 and 1,700 nm (Figure 2b). This behavior is likely due to the metallic properties of Ti_3_C_2_T_*x*_, which strongly influence the optical response in the infrared region. The interplay of transmission enhancement, reduced absorption, and increased reflection highlights the complex optical nature of mixed-MXene films. It also underscores the importance of further exploring compositional control in tailoring the films’ optical performance through VASE.

**Figure 3:**
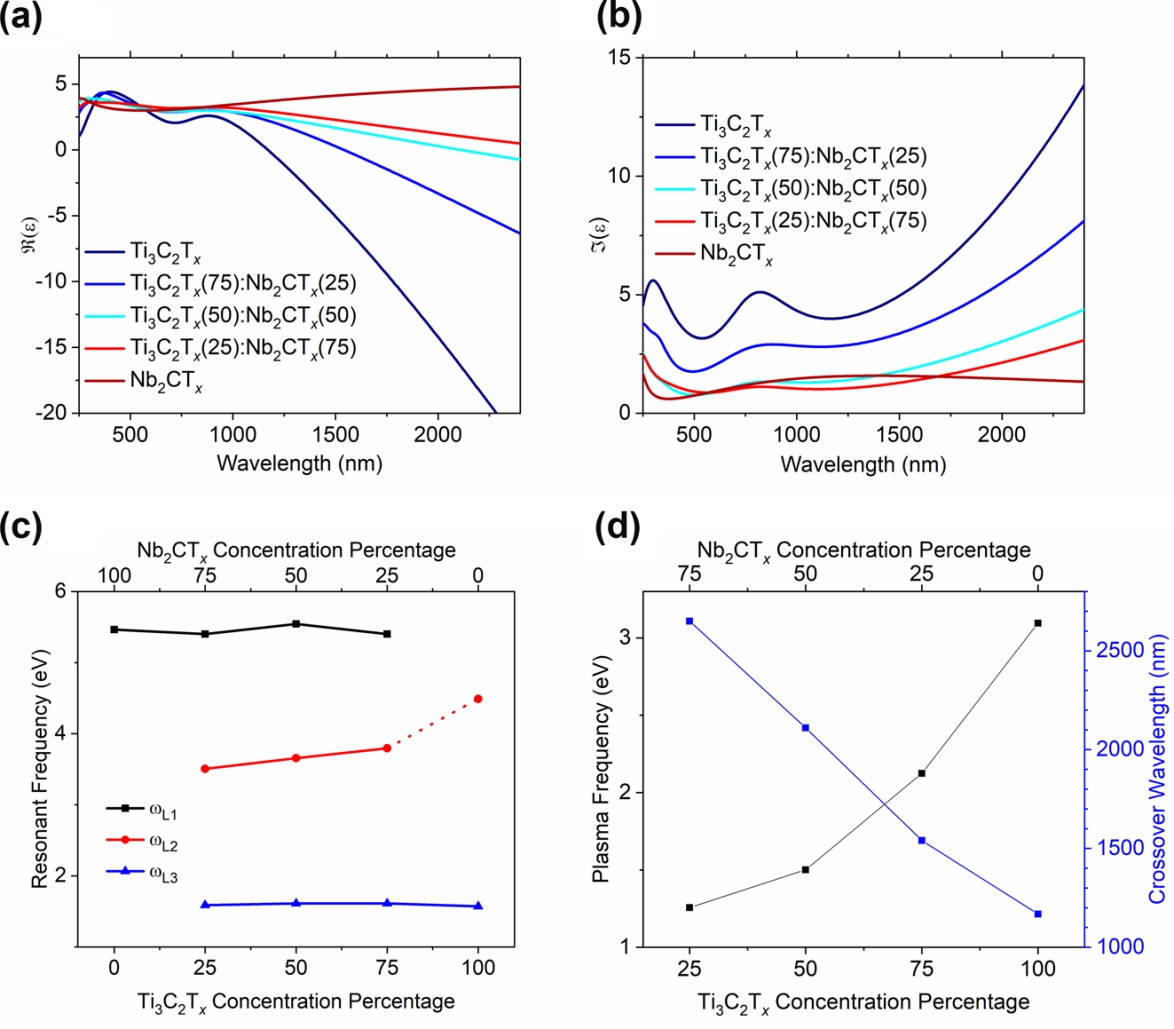
Dielectric permittivity values (a) real and (b) imaginary of Ti_3_C_2_T_*x*_ and Nb_2_CT_*x*_ mixed-MXene films. (c) Lorentz resonance frequencies plotted versus Ti_3_C_2_T_*x*_ concentration percentage. (d) Plasma frequency and the dielectric permittivity $ℜ\left(\varepsilon \right)$ zero-crossing point plotted versus Ti_3_C_2_T_*x*_ concentration percentage. Note that the *ω*_*L*2_ resonance is associated with a higher frequency Lorentz oscillator for the pure Ti_3_C_2_T_*x*_ film for consistency.

The Drude-Lorentz model was used to fit the VASE data and retrieve the real, $ℜ\left(\varepsilon \right)$ , and imaginary, $ℑ\left(\varepsilon \right)$ , components of the dielectric permittivity of the mixed-MXene thin films (Figure 3a–d). Both $ℜ\left(\varepsilon \right)$ and $ℑ\left(\varepsilon \right)$ fall between the pure titanium- and niobium-based films. The metallic optical properties indicated by the IR tail (Figure 3a) were parameterized in terms of the plasma frequency and ENZ wavelength (Figure 3d). Notably, the ENZ wavelength was in the SWIR spectrum and exhibited a substantial redshift of 1,500 nm as the Ti_3_C_2_T_*x*_ concentration decreased. The redshift was induced by the change in the plasma frequency of the films, which varied from 1.25 eV to 3.09 eV as a function of composition (Figure 3d). This result highlights the continuous tunability of the metallic properties of the films controlled by the mass composition. In contrast to the Ti_3_C_2_T_*x*_-based conducting films exhibiting Drude dispersion, pure Nb_2_CT_*x*_-based films are optically dielectric, with their NIR dispersion fitted by a heavily damped Lorentz, preventing the ENZ from occurring. The resonant frequencies of the Lorentzian fits, *ω*_*L*1_, *ω*_*L*2_, and *ω*_*L*3_, were similar across all samples (Figure 3c). The *ω*_*L*1_ resonance at approximately 5.4 eV appeared in niobium-containing films, while the *ω*_*L*3_ resonance at 1.6 eV and the *ω*_*L*2_ resonances ranging from 3.5–4.5 eV were seen in the titanium-containing films. The complete Drude–Lorentz oscillator parameters are provided in the Supplementary Material.

## 3 Discussion

The mixed-MXene films contained two different types of resonances represented by the Drude-Lorentz model. The Drude portion represents the effects of free-carrier plasma oscillations, while the Lorentz terms could signify interband or other optical transitions. Focusing first on the Lorentz terms, we note that the transition energies were maintained across films with different MXene ratios (Figure 3c). This consistency suggests that the influence of neighboring MXenes on the band structure was minimal, unlike in other 2D material systems such as graphene [28]. On the contrary, the Drude term represented by the plasma frequency was influenced by the MXene composition. The plasma frequency exhibited a blue shift as Ti_3_C_2_T_*x*_ increased, which we attribute to increasing carrier density according to the equation $\omega_{p}=\sqrt{N_{c}q^{2}/\varepsilon_{0}m^{*}}$ , where *N*_*c*_ is the free carrier density, *q* is the charge of an electron, *ɛ*_0_ is the dielectric permittivity of free space, and m* is the electron’s effective mass [27]. From this equation and the non-square-root dependence of the plasma frequency on the concentration, we can deduce that the electrons provided by the Ti_3_C_2_T_*x*_ are influenced by more complex interactions, such as charge transfer mechanisms between flakes or alternative interactions.

Notably, in the spectral region of approximately 900–1,400 nm, $ℑ\left(\varepsilon \right)$ of the mixed films of Ti_3_C_2_T_*x*_(50): Nb_2_CT_*x*_(50) and Ti_3_C_2_T_*x*_(25):Nb_2_CT_*x*_(75) had lower losses compared to the pure Nb_2_CT_*x*_ film (Figure 3b), and is observation was corroborated by the UV-VIS-IR absorption data (Figure 2c). The underlying reasons for this unexpectedly low loss in mixed-MXene films remain to be fully elucidated. Interestingly, we note that for the Ti_3_C_2_T_*x*_(25):Nb_2_CT_*x*_(75) film, the plasma frequency *ω*_*p*_, a Lorentz resonance *ω*_*L*3_, and the spectral window of decreased absorption all coincide, suggesting a synergistic interaction between the optically metallic Ti_3_C_2_T_*x*_ and dielectric Nb_2_CT_*x*_. Other considerations should include the packing density of the mixed films, the difference in flake size, and morphology [29], [30], [31]. Compared to Nb_2_CT_*x*_ flakes, the Ti_3_C_2_T_*x*_ flakes were more monolayered, and 5–10 times larger, which potentially improved the packing density and resulted in reduced loss due to scattering. Interestingly, as the concentration of Ti_3_C_2_T_*x*_ decreased in the film, the fine wire-like features also diminished (Figure 1b–f).

Interpreting the physical mechanisms behind tuning and reduced absorption requires further exploration into the complex electronic and optical environments of MXene flakes and films. In optical characterization, effects arising from mixed-termination MXenes, flake interactions, and polarizability dynamics due to disordered flake arrangements manifest as inhomogeneous broadening. Analyzing oscillator lineshapes presented in ellipsometry measurements could provide further insights. For example, modeling the absorption peaks with flexible lineshapes [32] revealed a stark difference in the nature of the Lorentzian peaks. The pure Ti_3_C_2_T_*x*_ films, where the *ω*_*L*3_ peak was purely Lorentzian, and *ω*_*L*2_ the peak had significant inhomogeneous broadening (see Supplementary Material). Although beyond the paper’s scope, the effects could be further resolved through monitoring carrier coherence dynamics with complex Fourier transform spectroscopy measurements [33].

The tunability and reduced loss of mixed-MXene films could support interesting optical applications. One exciting avenue of such characteristics is the control of the ENZ optical properties [25], [26], [27], [28], [29], [30], [31]. In pure Ti_3_C_2_T_*x*_, optical losses must be reduced for the strong manifestation of ENZ phenomena. We uniquely demonstrated that mixing an optically metallic and dielectric MXene reduced optical losses and simultaneously tuned the ENZ wavelength. In contrast, tuning the ENZ spectral range of organic materials by combining two distinct components often diminishes ENZ effects due to the disruptions in molecular aggregation [34]. Additionally, MXenes are known to have strong saturable absorption nonlinear optical properties in the infrared spectrum overlapping with important telecom and fiber laser operation wavelengths. A nonlinear enhancement due to the ENZ effects in mixed-MXene films could further enhance relevant technological applications.

Mixed-MXene films provide two knobs of tunability, where the first selects the Lorentz frequency through the choice of MXene, and the second controls plasma frequency – the Drude term – via the relative concentration of the two MXenes. Having two knobs for tunability in the mixed films enables easy optimization of resonance wavelengths. In contrast, tailoring solid solution MXenes through composition affects the position of the Lorentz resonances [17].

Future work includes optimizing the optical properties of mixed-MXene films iteratively by combining multiple MXenes and developing predictive models similar to Shrestha et al. [35]. Such models could incorporate compositional tuning, structural characteristics, and interfacial charge dynamics to physically understand and further enhance the performance of this novel composite material. By integrating experimental observations with advanced theoretical frameworks, the potential of mixed-MXene films as next-generation optical materials can be fully realized. This experimental study guides future research to explore additional material combinations, refine optical models, and advance the design of low-loss, tunable, and highly functional thin-film systems.

## 4 Conclusions

In conclusion, this study experimentally demonstrated a simple yet highly effective method to tailor the optical properties of MXene films by mixing MXenes with distinct compositions and features. We observed substantial tunability of the real part of the dielectric permittivity ENZ point ranging from 1.1 µm for a pure Ti_3_C_2_T_*x*_ film to 2.6 µm for the Ti_3_C_2_T_*x*_(25):Nb_2_CT_*x*_(75) film. The obtained ENZ point tunability exceeded previously reported ranges by over an order of magnitude, opening new possibilities for MXene applications by leveraging enhanced optical nonlinearities, such as the slow-light effect [23], [24], [36].

By systematically varying the relative concentrations of optically metallic Ti_3_C_2_T_*x*_ and dielectric Nb_2_CT_*x*_, we observed distinct and highly tunable optical behaviors. Notably, the optical losses within the films were significantly reduced with increased Nb_2_CT_*x*_ concentration, marking a substantial improvement in optical performance. This loss reduction was particularly noteworthy as high intrinsic losses in Ti_3_C_2_T_*x*_ have been a limiting factor for optical and plasmonic applications. Incorporating Nb_2_CT_*x*_ effectively mitigated this drawback, both preserving and potentially enhancing ENZ effects.

This proof-of-principle demonstration of mixed-MXene films represents a significant advancement, building upon the diverse optical properties offered by the extensive family of MXenes, including those in the IR range [36]. The straightforward methodology presented here opens new pathways for designing optical materials with unprecedented versatility and customization. The fabrication of mixed-MXene films uniquely leverages the simplicity of solution-based techniques to rapidly prepare and iterate samples with broad optical tunability. This capability contrasts with the synthesis-intensive approach required for solid-solution films, where each stoichiometry demands a distinct material preparation [17]. Our approach makes it possible to effectively “edit” the material’s optical properties for applications that exploit light–matter interactions, such as advanced optical modulators, sensors, and nonlinear optical devices. The simplicity of tailoring the dielectric permittivity, combined with the reduction in optical losses, makes these systems highly versatile for applications requiring precise control over optical properties.

## 5 Materials and methods

### 5.1 MXene synthesis

One gram of stoichiometric Ti_3_AlC_2_ MAX powder (produced following our previously reported synthesis protocols) [37] was slowly added to a 20 mL mixture of concentrated HF (49 wt%), concentrated HCl (36 wt%), and deionized water, with a volumetric ratio of 1:6:3. The MAX phase was etched at 35 °C for 24 h. After the reaction was completed, the etching product was washed with deionized water via multiple centrifugation steps (3,500 rpm, 5 min) until neutral pH. To delaminate the MXene sheets, the Ti_3_C_2_T_*x*_ multilayer product was stirred at 300 rpm in a 50 mL aqueous solution containing 1 g of LiCl at 35 °C for 18 h. Then, LiCl was removed from the solution by a single centrifugation step (3,500 rpm, 5 min), discarding the clear supernatant. After that, Ti_3_C_2_T_*x*_ was redispersed in water via shaking and centrifuged for 15 min at 3,500 rpm (to precipitate the multilayer MXene and unreacted MAX particles). Finally, the dark supernatant (containing single-layer MXene sheets) is collected.

One gram of Nb_2_AlC MAX powder was etched in a 20 mL mixture of HCl and HF, with a volume ratio of 12:8 at 48 °C for 50 h. After etching, the solution was repeatedly centrifuged with DI water at 3,500 rpm for 2 min until pH > 6. The obtained sediment was collected for delamination. The MXene flakes were delaminated using tetramethylammonium hydroxide (TMAOH). In particular, 5 mL of 25 wt% TMAOH solution was added to 1 g of etched MXene and stirred for 12 h at 35 °C. After that, the mixture was repeatedly centrifuged with DI water at 3,500 rpm for 10 min until pH < 8. Following delamination, the MXene dispersion was concentrated via high-speed centrifugation at 10,000 rpm.

### 5.2 Spray coating of film

Before use, the fused silica substrates were cleaned in a sonication bath in acetone and 2-propanol for 10 min each. After that, they were heated on a hot plate at 60 °C. Then, Ti_3_C_2_T_*x*_, Nb_2_CT_*x,*_ and their mixtures (aqueous dispersions with a concentration of ≈0.5 mg/mL) were sprayed using a commercial airbrush gun with a needle and nozzle diameter equal to 0.3 mm. The distance between the nozzle tip and the substrate was kept at about 20 cm. We describe the mixed films by Ti_3_C_2_T_*x*_(Y):Nb_2_CT_*x*_(100-Y), where Y is the mass percentage of Ti_3_C_2_T_*x*_ in the film.

### 5.3 Optical characterization

One mixed-MXene film was characterized for each concentration. We employed two standard techniques to characterize the optical properties of the fabricated thin films. Transmission and reflection spectra were measured with a Perkin Elmer Lambda 950 UV-VIS-NIR spectrometer. The subsequently calculated absorption was then used to identify optical transitions. The complex refractive index and permittivity were retrieved via spectroscopic ellipsometry (J. A. Woollam R2C VASE). Data was collected over a broad spectral (210–2,500 nm) and angular (55–75°) range to ensure unique fitting. The data was fitted alongside the transmission spectrum, and optical parameters were extracted with the CompleteEASE software.

## Supplementary Material

Supplementary Material Details
