# Supplementary material for: Tailoring optical response of MXene thin films
Source: Nanophotonics. 2025 Apr 3;14(23):4091–7. doi: 10.1515/nanoph-2024-0769 (PMC12617701; doi:10.1515/nanoph-2024-0769)
Supplement: Supplementary file 1 — Supplementary Material Details [file j_nanoph-2024-0769_suppl_001.pdf]

Supplementary Material

Tailoring Optical Response of MXene Thin Films

Jeffrey Simon[1†], Kyu Ri Choi[1,2†], Stefano Ippolito[3], Ludmila Prokopeva[1], Colton Fruhling[1], Vladimir M. Shalaev[1], Alexander V. Kildishev[1], Yury Gogotsi[3], and Alexandra Boltasseva[1,4*]

1Elmore Family School of Electrical and Computer Engineering, Birck Nanotechnology Center and Purdue Quantum Science and Engineering Institute, Purdue University, West Lafayette, IN, 47907, USA

2 Research Institute for Nanoscale Science & Technology, Chungbuk National University, Cheongju, Chungbuk 28644, Republic of Korea

3 A. J. Drexel Nanomaterials Institute and Department of Materials Science and Engineering, Drexel University, Philadelphia, PA, 19104, USA

4 School of Materials Engineering, Purdue University, West Lafayette, IN 47907, USA

[†]These authors contributed equally to this work

** Email: aeb@purdue.edu*

Contents

S1. Film Characterization.. 2

S2. Drude-Lorentz Oscillator Parameters.. 4

S3. Inhomogeneous Broadening Study.. 8

Reference.. 8

S1. Film Characterization

Table S1: Morphological properties of mixed-MXene films measured via AFM

Film	Thickness (nm)	RMS Roughness (nm)
**$Ti_3C_2T_x$**	16.8 ± 1.8	14.6
**$Ti_3C_2T_x$(75):Nb_2CT_x(25)**	20.8 ± 0.6	14.8
**$Ti_3C_2T_x$(50):Nb_2CT_x(50)**	29.1 ± 0.2	12.9
**$Ti_3C_2T_x$(25):Nb_2CT_x(75)**	29.2 ± 0.3	13.8
**Nb_2CT_x**	34.8 ± 1.8	8.8

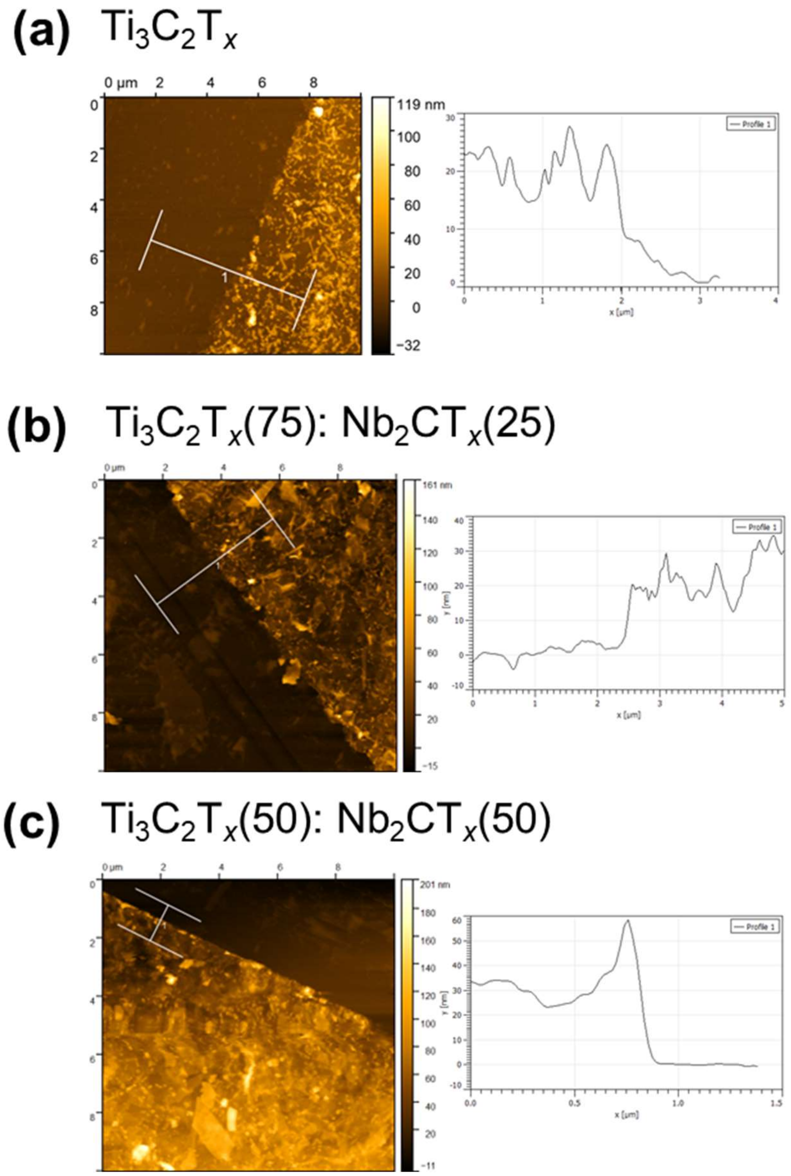

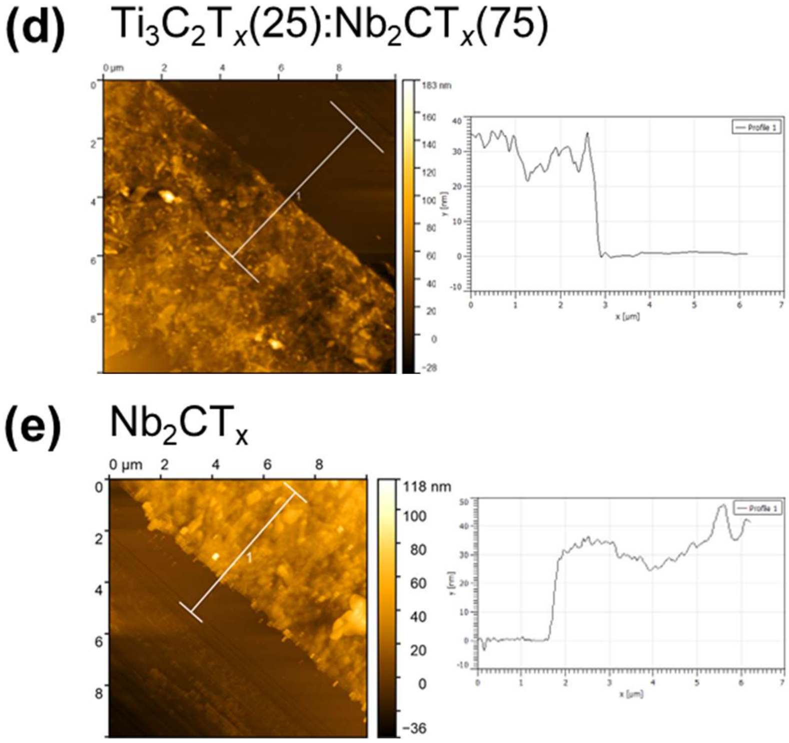

Figure S1: (a-e) AFM images and corresponding thickness profiles of the $Ti_3C_2T_x$ and Nb_2CT_x mixed-MXene samples.

S2. Drude-Lorentz Oscillator Parameters

The Drude-Lorentz (DL) models for all $Ti_3C_2T_x$ concentrations were successfully extracted using the following three-step ellipsometry fitting process. In Step 1, the likely dielectric function was extracted using Kramers-Kronig consistent B-splines under a nonnegative absorption constraint. In Step 2, the DL models were fitted to the B-spline solution to provide the search algorithms with a reliable initial guess. In Step 3, the Drude-Lorentz models were refined through direct fitting to the ellipsometry parameters, the amplitude component Ψ and the phase difference Δ, yielding a range of the mean-square errors (MSEs) from 7 to 13 for $Ti_3C_2T_x$ concentrations from 0 to 100%, respectively, across the visible to near-infrared spectrum. Figure S2 presents the pivoting B-spline solutions and final extracted DL dielectric functions for pure films for $Ti_3C_2T_x$ and Nb_2CT_x,

$$\varepsilon(\omega) = \varepsilon_\infty + \frac{f_{\mathrm{UV}}}{\omega_{\mathrm{UV}}^2 - \omega^2} - \frac{\omega_{\mathrm{p}}^2}{\omega^2 + i\Gamma_{\mathrm{D}}\omega} + \sum_i \frac{f_L \omega_{Li}^2}{\omega_{Li}^2 - \omega^2 - i\Gamma_{Li}\omega} \tag{S2.1}$$

Table S2: Parameters of the Drude-Lorentz models extracted for $Ti_3C_2T_x$ and Nb_2CT_x mixed-MXenes.

	Thickness (nm)	High-Frequency permittivity	UV Pole		Drude		Lorentz		
		ε_∞	f_{UV}	ω_{UV} (eV)	ω_{p} (eV)	Γ_{D} (eV)	ω_L (eV)	Γ_L (eV)	f_L
$Ti_3C_2T_x$	13 ± 0.49	1.000	46.359	7.210	3.096	0.224	4.488<br>1.570	3.431<br>0.845	4.056<br>1.731
$Ti_3C_2T_x$(75): Nb_2CT_x(25)	30 ± 0.32	1.021	49.918	7.626	2.124	0.312	5.401<br>3.795<br>1.611	3.450<br>1.269<br>1.353	2.227<br>0.289<br>1.588
$Ti_3C_2T_x$(50): Nb_2CT_x(50)	30 ± 0.23	1.387	44.991	7.561	1.501	0.435	5.543<br>3.656<br>1.611	2.820<br>1.408<br>1.118	1.331<br>0.102<br>0.557
$Ti_3C_2T_x$(25): Nb_2CT_x(75)	29 ± 0.27	1.714	21.774	7.022	1.256	0.415	5.403<br>3.506<br>1.587	2.595<br>2.716<br>1.025	1.130<br>0.379<br>0.379
Nb_2CT_x	25 ± 0.46	1.858	25.039	6.834	N/A	N/A	5.464<br>1.572	1.702<br>3.257	0.602<br>2.428

ε_∞: high-frequency background

f_{UV}: UV pole strength

ω_{UV}: UV pole frequency

ω_p: Drude plasma frequency

Γ_{D}: Drude damping

f_L: strength

ω_L: resonant frequency

Γ_L: broadening of Lorentz oscillator

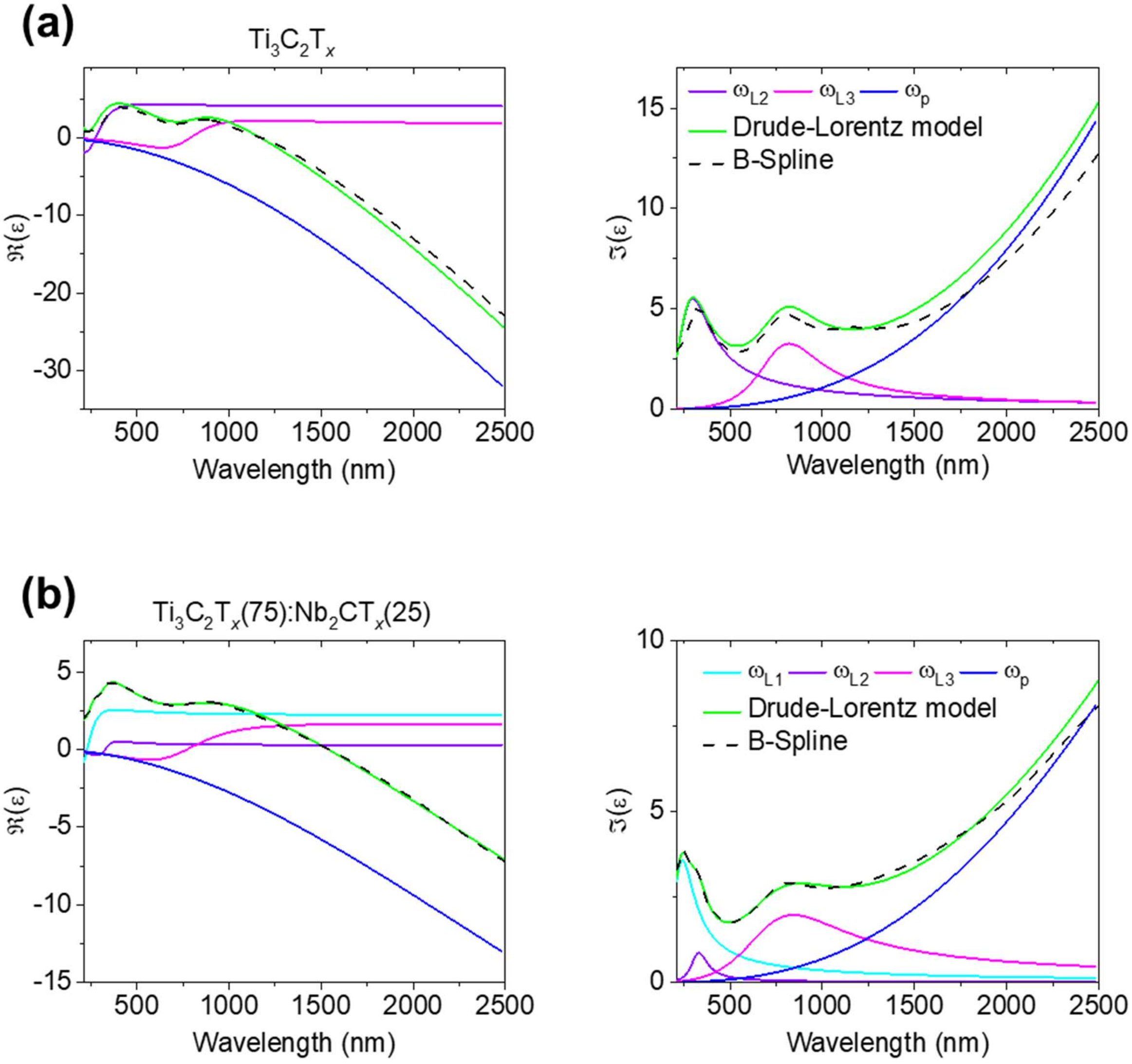
(a)
Ti3C2Tx
ℜ(ε)
Wavelength (nm)
ωL2
ωL3
ωp
Drude-Lorentz model
B-Spline
ℑ(ε)
Wavelength (nm)
(b)
Ti3C2Tx(75):Nb2CTx(25)
ℜ(ε)
Wavelength (nm)
ωL1
ωL2
ωL3
ωp
Drude-Lorentz model
B-Spline
ℑ(ε)
Wavelength (nm)

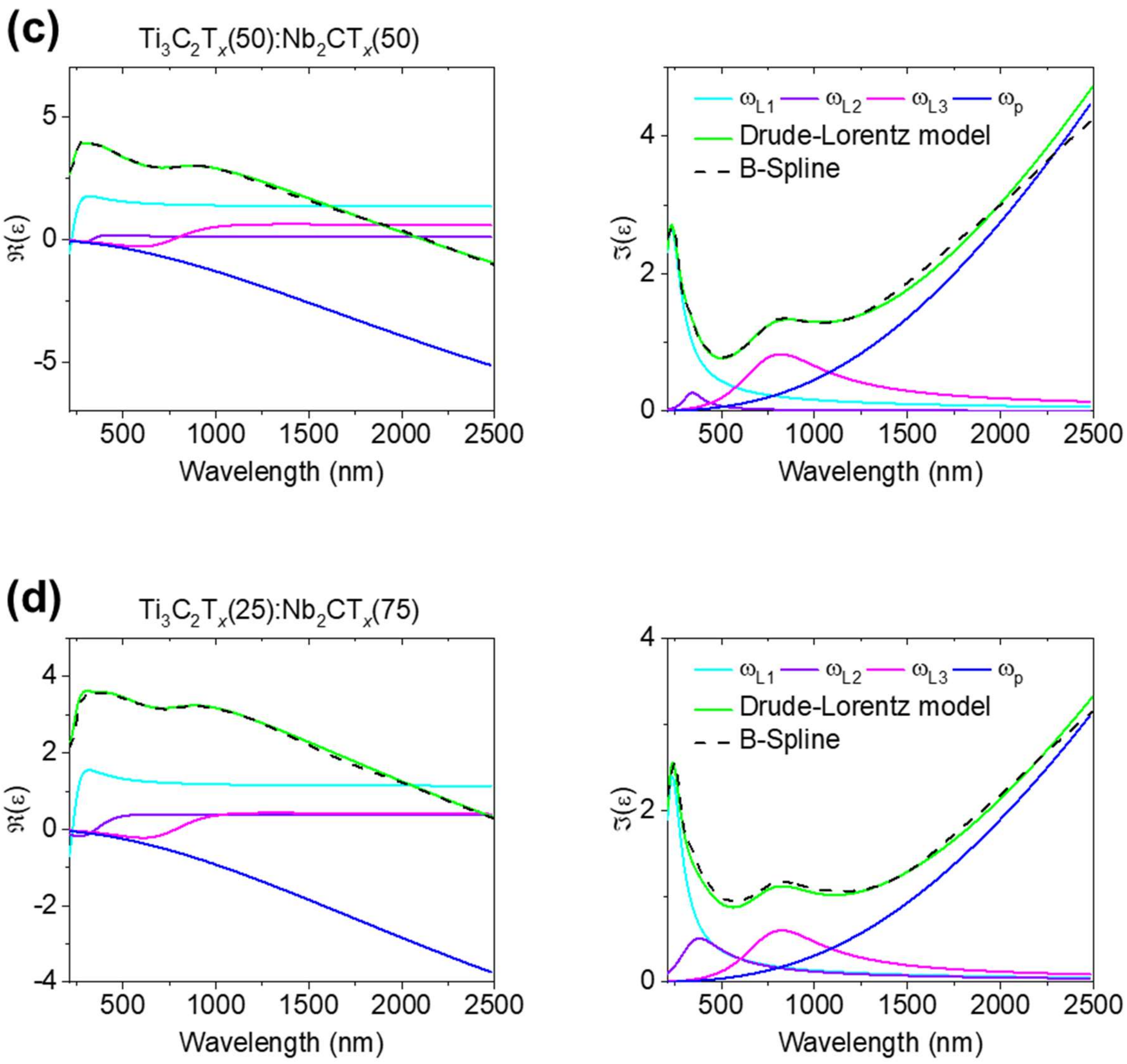
(c)
Ti3C2Tx(50):Nb2CTx(50)
ℜ(ε)
Wavelength (nm)
ℑ(ε)
ωL1
ωL2
ωL3
ωp
Drude-Lorentz model
B-Spline
(d)
Ti3C2Tx(25):Nb2CTx(75)
ℜ(ε)
Wavelength (nm)
ℑ(ε)
ωL1
ωL2
ωL3
ωp
Drude-Lorentz model
B-Spline

(e)

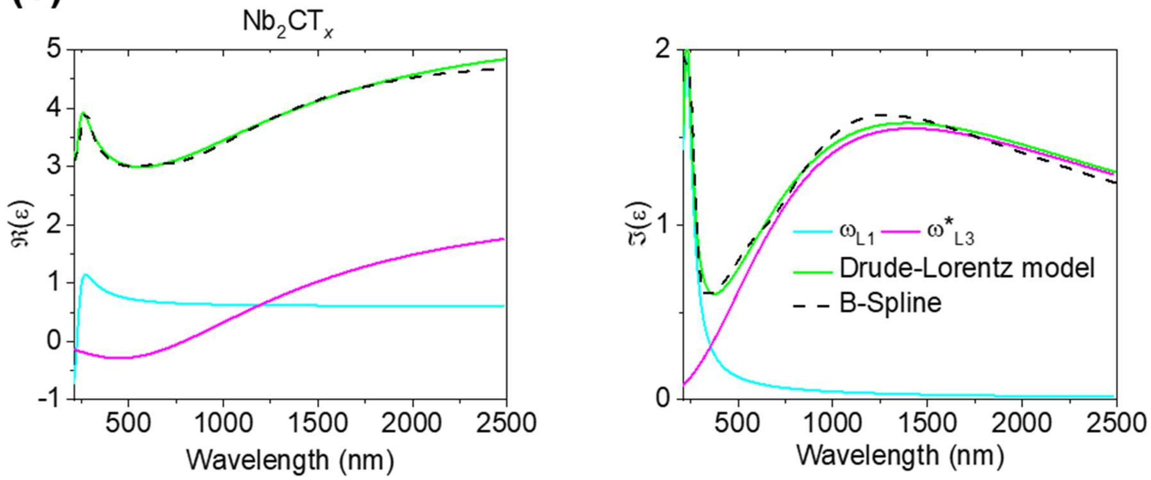

Figure S2: B-spline and Drude-Lorentz model with oscillators ω_{L1} , ω_{L2} , ω_{L3} , and ω_p (a-e) for corresponding concentration of $Ti_3C_2T_x$ and Nb_2CT_x. Note that ω_{L3}^* in (e) represents the heavily damped oscillator.

S3. Inhomogeneous Broadening Study

To investigate inhomogeneous broadening, the Drude-Lorentz model was augmented with additional parameters (DL-I model) [1]. Our investigation focused on pure $Ti_3C_2T_x$ where the resonances ω_{L2} and ω_{L3} are the strongest and are in the range of the variable angle spectroscopic ellipsometry (VASE) data. This analysis revealed that the ω_{L3} peak was purely Lorentzian and only had homogeneous broadening. In contrast, the ω_{L2} peak exhibited a salient spectral diffusion around the Lorentzian component (shown as the filled center peak in Figure S3(b)) resulting in a significant inhomogeneous broadening. When the broadening is homogeneous (defined by finite lifetime), the resonance is expected to remain independent of $Ti_3C_2T_x$ concentration. On the contrary, inhomogeneous broadening would cause resonance drift. This could explain why ω_{L3} in Figure 3(c) of the main text remains fixed across all concentrations, while ω_{L2} varies. More advanced models that provide better accuracy and insight into the statistical characterization of spectral diffusion and resonance drifts will be the subject of further investigations.

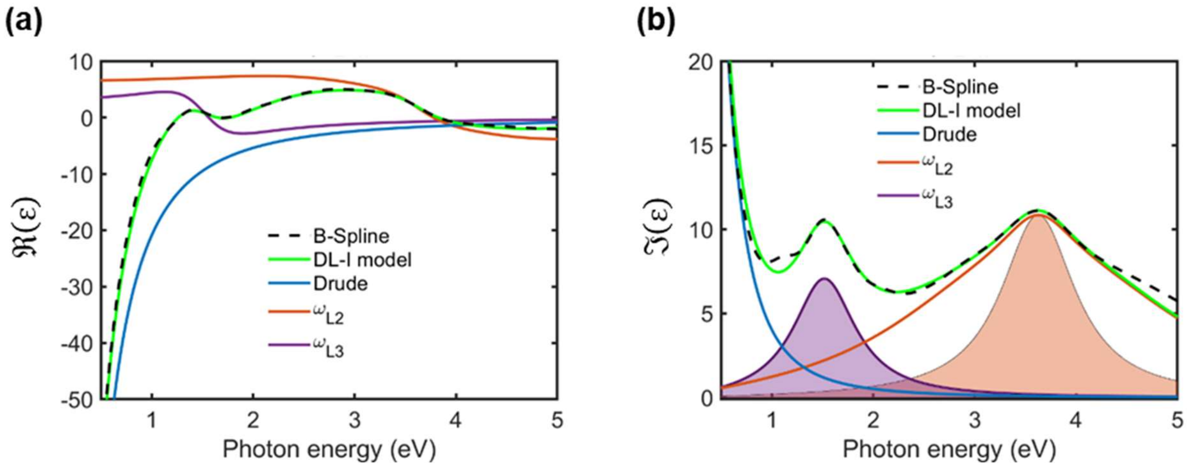

Figure S3: (a) Real and (b) imaginary permittivity components of the decomposition of absorption peaks ω_{L2}, and ω_{L3} in pure $Ti_3C_2T_x$ film into homogeneous (Lorentzian) and inhomogeneous (non-Lorentzian) components. Lorentzian parts (shown as filled peaks) are intrinsic to the material and remain spectrally stable, while inhomogeneous broadening depends on flake morphology as well as distribution, and it varies with concentration.

Reference

[1] L. J. Prokopeva, S. Peana, and A. V Kildishev, “Gaussian dispersion analysis in the time domain: efficient conversion with Padé approximants,” *Comput. Phys. Commun.*, vol. 279, p. 108413, 2022, doi: 10.1016/j.cpc.2022.108413.
